# Pediatric ventriculoperitoneal shunt: a comparative study between anterior fontanel ultrasound-guided versus conventional cranial end insertion

**DOI:** 10.1007/s00381-022-05807-x

**Published:** 2022-12-23

**Authors:** Wael Abd Elrahman Ali Elmesallamy, Akrem Mhemed Ali Abofaid, Mohamed Salah Mohamed, Mahmoud M. Taha

**Affiliations:** 1 Faculty of Human Medicine, Zagazig University, Zagazig, Alsharkia Egypt; 2 Faculty of Human Medicine, Tripoli University, Tripoli, Libya

**Keywords:** Pediatric hydrocephalus, Ventriculoperitoneal shunt, Trans-anterior fontanel ultrasound, Proximal part location

## Abstract

**Purpose:**

Ventriculoperitoneal (V-P) shunt is one of the most common neurosurgical procedures in pediatrics for the treatment of hydrocephalus. Shunt failure is one of the common mechanical complications which lead to major morbidities. This study aims to compare between cranial part insertions of the V-P shunts guided by trans-anterior fontanel ultrasound versus conventional insertion.

**Methods:**

A prospective comparative randomized study was conducted on 60 pediatric patients aged ≤ 2 years who suffered hydrocephalus and allocated into 2 groups. In the first group (*n* = 30), the cranial parts of the ventriculoperitoneal shunts were inserted guided by trans-anterior fontanel ultrasound, and in the second group (*n* = 30), the insertions were by the conventional method. The follow-up duration of the patients was 3 months.

**Results:**

Proximal part obstruction of the V-P shunt was found in 3 cases of the conventional group during follow-up with statistical insignificance (*p* = 0.237) while adequate proximal part location recorded statistical significance (*p* = 0.0005) in favor of ultrasound-guided group.

**Conclusion:**

The use of the anterior fontanel ultrasound guide during ventriculoperitoneal shunt insertion is a feasible, safe, and effective technique for the placement of ventricular catheters in pediatric patients with a patent anterior fontanel.

## Introduction

The under-tension accumulation of cerebrospinal fluid (CSF) in the brain ventricles and subarachnoid spaces is known as hydrocephalus; hydrocephalus is a result of abnormalities in the secretion or absorption process of CSF [1]. The incidence of hydrocephalus is high in poor countries (123 per 100,000 births) than in high-income countries (79 per 100,000 births) [2]. Ventriculoperitoneal shunts are among the most common procedures performed by neurosurgeons as a surgical treatment for hydrocephalic patients, but cerebrospinal fluid shunt failure is related to additional morbidity. Misplacement of ventricular catheters occurs in 40% with freehand technique and is a risk factor for shunt failure [3]. Even with the truth that the incorrect insertion of a ventricular catheter may lead to disastrous consequences, most surgeons are still using “blind” conventional technique catheter placement [4]. With the conventional technique of ventriculoperitoneal shunt catheter insertion, the selection of the site of entry and the pathway is based on the anatomical landmarks and preoperative imaging, and the experience of the surgeon [5]. The misplaced ventricular catheter may lead to disastrous neurological morbidity and it has a high risk of obstruction in contrast to that placed well in the right position [6]. Intraoperative ultrasound can explore the anatomy of the lateral ventricles and the choroid plexus before catheter insertion. Also, determine the distance and trajectory to the better location. The catheter is visualized and inserted during real-time ultrasound monitoring [7].

The aim of this study is to highlight the effectiveness of cranial part insertion of the V-P shunt guided by trans-anterior fontanel ultrasound in comparison to the conventional method.

## Methods

A comparative randomized prospective study included 60 pediatric patients admitted to our neurosurgery department, at our academic university hospital. They were suffering from hydrocephalus and were subjected to ventriculoperitoneal shunt during the period from January 2020 to January 2022.

Written informed consents were obtained from all parents of the children and the study was approved by our Institutional Review Board (IRB) [IRB#:5782–1-12–2019] in accordance with The Code of Ethics of the World Medical Association (Declaration of Helsinki) for studies involving humans.

### Inclusion criteria

Patients ≤ 2 years old suffering from hydrocephalus with open anterior fontanel, peripheral cerebral brain mantel ≥ 2 CM, head circumference ≤ 50 CM, and without previous shunt insertion or intraventricular septation.

### Interventional procedures

The patients were divided randomly into 2 groups. In the first group (*n* = 30), the cranial parts of the V-P shunts were inserted guided by trans-anterior fontanel ultrasound IBE-2500D scanner with endo-cavitary transducer 5–8 MHZ of footprint 18 × 8 mm. The transducer and acoustic gel were contained inside a surgical glove finger under an aseptic manner. The transducer could be used in different positions and angles at the opened anterior fontanel to see the brain and the ventricular system including the choroid plexus. The choroid plexus must be visualized and when targeting the frontal horn; our novel technique was tilting the transducer from coronal orientation with visualization of the catheter inside the ventricle to find the pathway beside the choroid plexus and continuation of the shunt pathway to the target location. The targets were either the lateral ventricular body away from the choroid plexus or the frontal horn of the lateral ventricle near the foramen of Monro.

In the second group (*n* = 30), the cranial parts of the V-P shunts were inserted by the conventional method.

The entry skull point was Keen point (3–4) CM. superior and (3–4) CM posterior to the helix of the ear) on the right side. Scalp incisions were in the shape of inverted U or C caring distances from valve edges, the periosteal layer was kept for valve securing by stitching, and the dura matter was cauterized by bipolar at the site of insertion puncture which was done by 11 or 15 sized scalpels. The proximal part should target the ipsilateral body away from the choroid plexus or the frontal horn. Cerebrospinal fluid is sent for cytological analysis and culture whenever suspected for abnormality. All operations were done under general anesthesia in a supine position with head tilt to the left side, the antibiotic was administrated intra-operatively, the skin was prepped by petadine surgical solution, and the shunt was not opened until it is needed and handled with new gloves. The distal catheter was tunneled from down to up through subcutaneous tissues and secured to the valve by a knot which must face toward the skull to avoid scalp irritation and the peritoneal end was inserted after confirmation of CSF flow. All patients were operated by medium-pressure shunts of size 12 Mm.

The follow-up duration was up to 3 months by:

1. Clinical evaluation:
  - Anterior fontanel: tense or lax
  - Valve condition: well functioning or delayed filling > 30 s or stony hard
  - Complications: such as CSF leak and infection
  - Head circumference
2. Imaging evaluation: with computed tomography scan (CT scan) within 48 h from surgery and during the follow up including:
  - Position of the ventricular catheter
  - Cerebral brain mantle thickness (paraventricular cerebral mantle)
  - Frontal horn diameter change
  - Periventricular edema changes
  - Complications: such as hemorrhage and infection

### Statistical analysis

All data were collected, tabulated, and statistically analyzed using IBM Corp. Released 2015. IBM SPSS Statistics for Windows, Version 23.0. Armonk, NY: IBM Corp. Quantitative data were expressed as the mean ± SD and qualitative data were expressed as number and (percentage). *T*-test was used to compare between two groups of normally distributed variables. The percentage of categorical variables was compared using chi-square test or Fisher’s exact test when appropriate. All tests were two-sided. *p*-value < 0.05 was considered statistically significant, and *p*-value ≥ 0.05 was considered statistically insignificant.

## Results

The attainable results of the two groups (the ultrasound-guided group and conventional group); showed the mean age by months was distributed as 5.89 ± 1.87 and 7.02 ± 2.31, respectively, with no significant difference between groups. There was no significant difference regarding sex distribution. No significant differences were found between the two groups regarding history distribution and examinations (Table 1). Concerning preoperative imaging, there was no significant difference between the two groups. Regarding postoperative imaging either within the first 48 h after surgery and after 3 months; the only recorded statistical significance was an adequate proximal part location in favor of the ultrasound group (Table 2); we excluded the re-operated cases during follow-up (3 cases operated for proximal obstruction and 3 cases operated for distal obstruction) as an initial surgical failure. The intent of this study was to evaluate the primary surgeries and their effect on the patients but not evaluate the patients in general, so after the failure of the primary surgery (6 patients), we decided to exclude those patients as they were subjected to another surgeries either revision of the peritoneal end or revision of the cranial end (all 3 patients were from the conventional group, but revisions were done sonar-guided). There was no significant difference found between the two groups regarding postoperative complication during the first postoperative month (Table 3). The operative time was calculated with a range 45 to75 min in both groups.

**Table 1:** Demographic data, history, and clinical examination between studied groups

**Variables**	**Ultrasound-guided group (*n*** **= 30)**	**Conventional group (*n*** **= 30)**	***t*/*X***^2^	***p***
Age (months)	5.89 ± 1.87	7.02 ± 2.31	2.08	0.041
**Sex**				
Male	16 (53.3%)	18 (60.0%)	0.271	0.602
Female	14 (46.7%)	12 (40.0%)		
**History**				
• Consanguinity	6 (20%)	6 (20%)	-	-
• Other family members with hydrocephalus	2 (6.7%)	3(10%)	f	0.99
• Maternal hazards exposure during pregnancy	7 (23.3%)	5 (16.7%)	0.416	0.518
• Intrauterine diagnosis	22 (73.3%)	18 (60%)	1.2	0.274
• CSF infection	2 (6.7%)	2 (6.7%)	-	-
• Intracranial hemorrhage	2 (6.7%)	6 (20%)	fisher	0.254
**Clinical Examination**				
**Other anomalies**				
• neurogenic	5 (16.7%)	7 (23.3%)	0.416	0.518
• Non neurogenic	6 (20.0%)	4 (13.3%)	0.48	0.488
**Head circumference**				
• ≤ 45 CM	12 (40.0%)	10 (33.3%)	0.287	0.592
• > 45 – < 50 CM	18 (60.0%)	20 (66.7%)		
**Anterior fontanel width**				
4—6 CM	20 (66.7%)	18 (60.0%)	0.287	0.591
> 6—10 CM	10 (33.3%)	12 (40.0%)	0.372	0.541
Bulging	22 (73.3%)	24 (80.0%)		
**Sunset eyes**	8 (26.7%)	12 (40.0%)	1.2	0.274
**Congested scalp veins**	15 (50.0%)	17 (56.7%)	0.267	0.604
**Craniofacial disproportion**	21 (70%)	24 (80.0%)	0.8	0.371
**Delayed milestone signs**	22 (73.3%)	24 (80.0%)	0.372	0.541

Data were expressed by either mean ± SD or number (%). *n*, the number of patients in each group. *N*, the number of patients in each category. *p* > 0.05, non-significant. *χ*^*2*^, chi-square test

**Table 2:** Preoperative and postoperative imaging data between studied groups

**Variables**	**Ultrasound-guided group**	**Conventional group**	***X***^**2**^	***p***
	***N* = 30, (%)**	***N* = 30, (%)**		
**Type of hydrocephalus**				
• Obstructive (non-communicating)	22 (73.3%)	20 (66.7%)	0.317	0.573
• Non-obstructive (communicating)	8 (26.7%)	10 (33.3%)		
**Corpus callosum agenesis**	6 (20.0%)	10 (33.3%)	1.36	0.243
**Other lesions**	2 (6.7%)	6 (20.0%)	f	0.254
**Trans ependymal edema**				
Preoperative	8 (26.7%)	10 (33.3%)	0.317	0.573
Postoperative				
• Within 48 h (same as preoperative)	8/8 (100%)	10/10 (100%)	-	-
• After 3 months (same as preoperative	5/8 (62.5%)	5/10 (50%)	-	-
**Maximal frontal horns width**				
Preoperative				
• 0.5–1 CM	6 (20.0%)	10 (33.3%)	1.36	0.243
• > 1 CM	24 (80.0%)	20 (66.7%)		
Postoperative				
• Within 48 h (same as preoperative)	30 (100%)	30 (100%)	0	1
• After 3 months (same as preoperative)	10/28 (35.7%)	10/26 (38.5%)	0.043	0.834
**Maximal cerebral mantle width opposite the body of lateral ventricle**				
Preoperative				
• 2–3 CM	22 (73.3%)	20 (66.7%)	0.317	0.573
• > 3 CM	8 (26.7%)	10 (33.3%)		
Postoperative				
• Within 48 h (same as preoperative)	30 (100%)	30 (100%)	0	1
• After 3 months (same as preoperative)	10/28 (35.7%)	10/26 (38.5%)	0.043	0.834
**Postoperative Ventricular tube**				
**Within 48 h**				
• Adequate	30 (100%)	18 (60%)		
• Short	0 (0%)	8 (26.7%)		
• Long, crossing the other side	0 (0%)	4 (13.3%)	15	0.0005
**After 3 months**				
• Adequate	27/28 (96.4%)	18/26 (64.3%)	7.73	0.021
• Short	0 (0%)	5 (19.2%)		
• Long, crossing the other side	1 (3.6%)	3 (11.5%)		

Data were expressed by number (%). *n*, the number of patient in each group. *N*, the number of patients in each category. *p* > 0.05, non-significant. *χ*^*2*^, chi-square test. *f*, Fisher’s exact test

**Table 3:** Operative complications during first postoperative month

**Variables**	**Ultrasound-guided group (*n* = 30)**	**Conventional group (*n*** **= 30)**	***X***^**2**^	***p***
**Tense fontanel**	2 (6.7%)	4 (13.3%)	f	0.67
**CSF leak**	1 (3.3%)	2 (6.7%)	f	0.99
**Proximal obstruction**	0 (0%)	3 (10%)	f	0.237
**Distal obstruction**	2 (6.7%)	1 (3.3%)	f	0.99
**Shunt infection**	2 (6.7)	2 (6.7)	f	1
**Intracranial hemorrhage**	0 (0%)	2 (6.7%)	f	0.491
**Total complicated cases**	6 (20%)	8 (26.7%)	0.48	0.488

Data were expressed by number (%). *n*, the number of patient in each group. *N*, the number of patient in each category. *p* > 0.05, non-significant. *χ*^*2*^, chi-square test

**Fig. 1:**
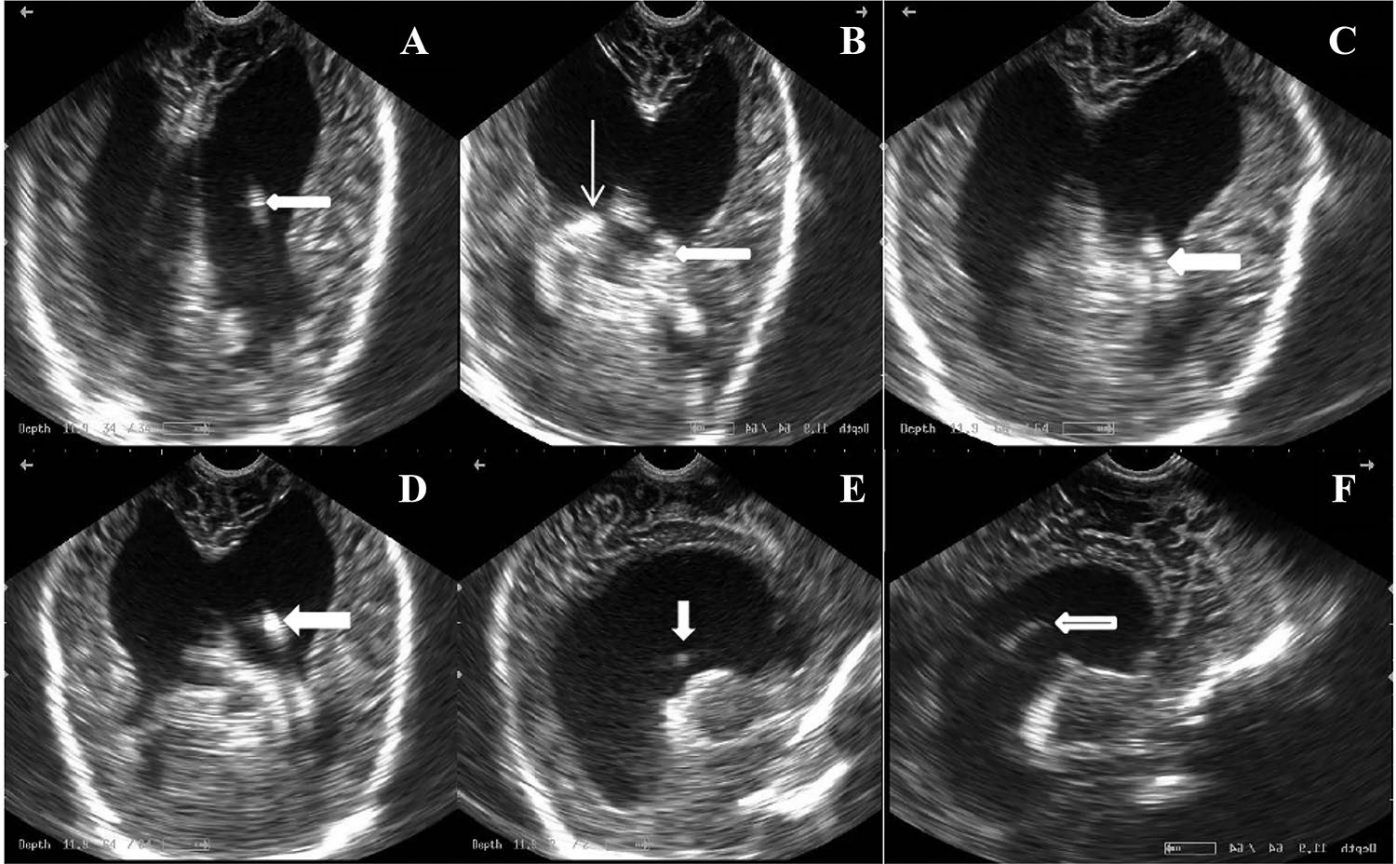
Steps of ultrasound guided cranial part insertion in the fontal horn; thick arrows point to the catheters and thin arrow points to the choroid plexus. **A** The catheter with the stylet in the body of the lateral ventricle, **B** identification of the choroid plexus, **C** finding the pathway to the frontal horn, **D** advance the catheter with the stylet to the desired level, **E** and **F** sagittal ultrasound views

**Fig. 2:**
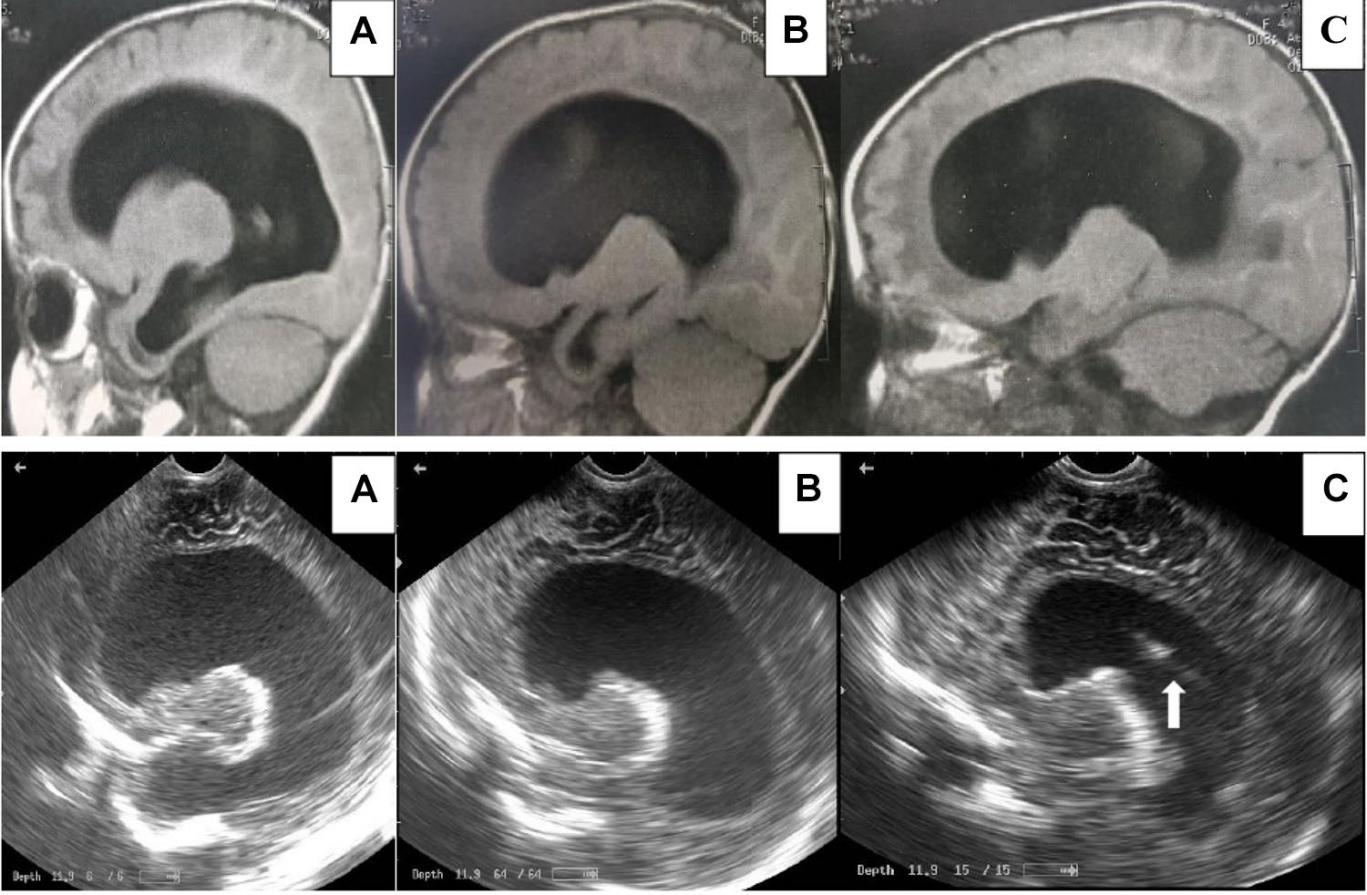
The ability of ultrasound to capture images at different sagittal levels equivalent to an MRI of the same patient. Note the catheter ( arrowed) in the last ultrasound image C

**Fig. 3:**
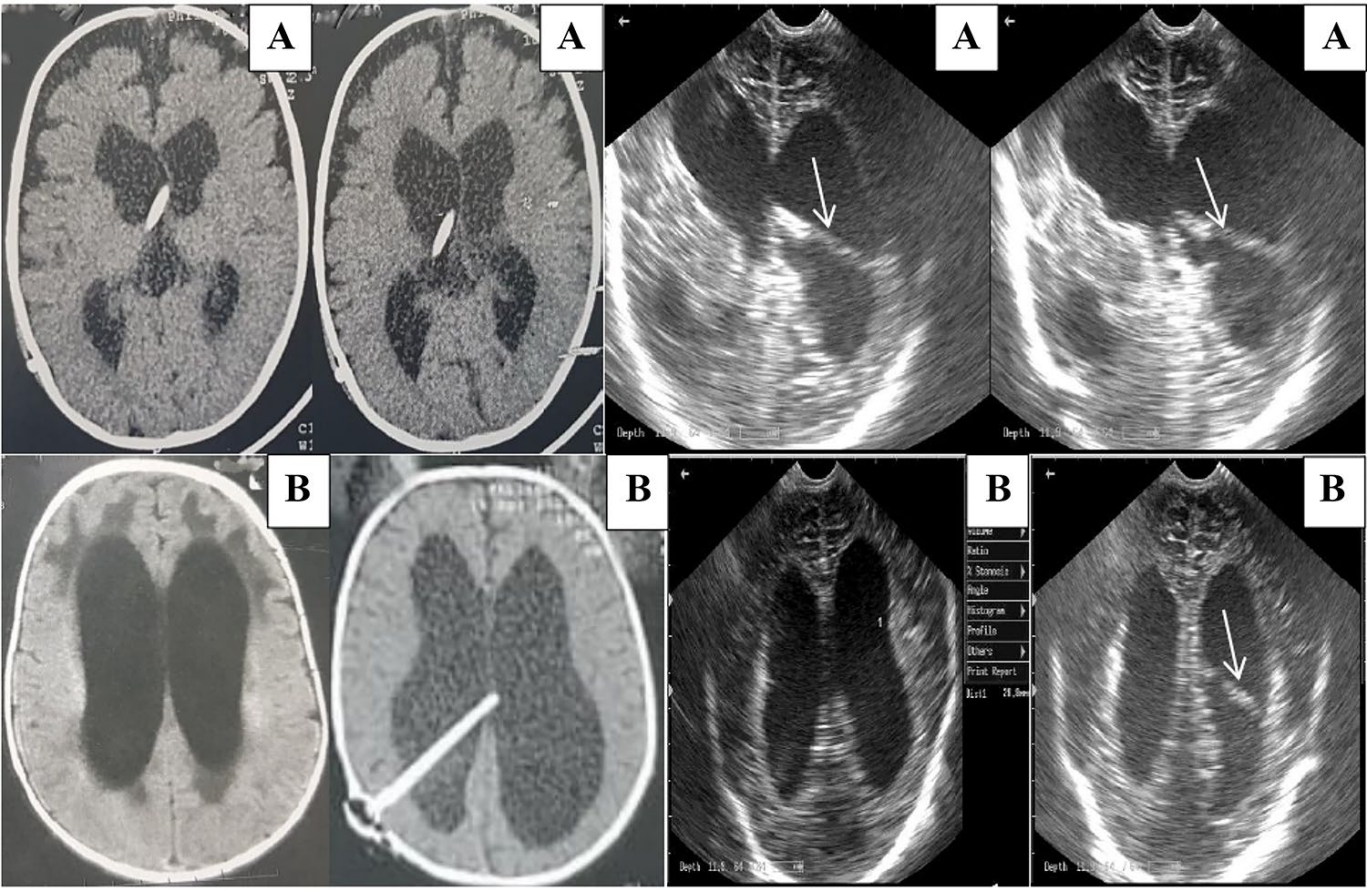
Equivalent CT and ultrasound images. Case A, with a catheter inside the frontal horn of the lateral ventricle. Case B, CT and ultrasound images before and after catheter insertion inside the body of the lateral ventricle. Thin arrows refer to the catheter inside the ventricles

**Fig. 4:**
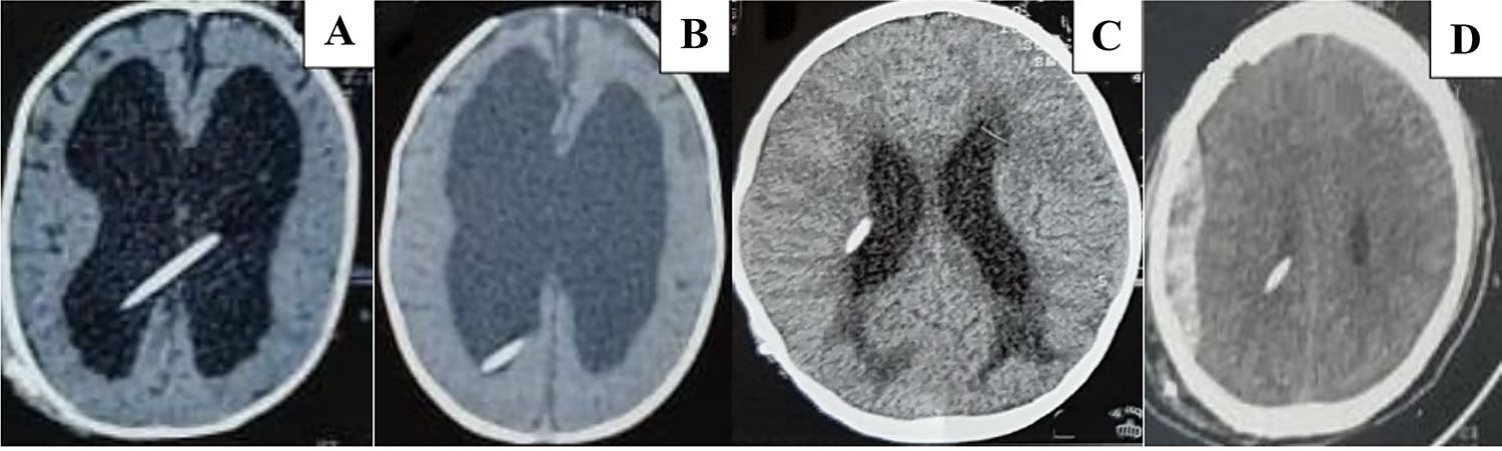
Inappropriate location and complications; long crossing tube (**A**), short tubes (**B**) and (**C)**. Extradural hematoma (**D)**

Figure 1 shows the steps of ultrasound-guided cranial part insertion in the fontal horn and demonstrates the novel technique; A: the tube with the stylet in the body, B: identification of the choroid plexus, C: finding the pathway to the frontal horn, D: advance the tube with the stylet to the desired level, E and F: sagittal ultrasound views. Figure 2 shows the ability of ultrasound to capture images at different sagittal levels equivalent to MRI. Note the tube in the last ultrasound view. Figure 3 shows equivalent CT and ultrasound images with a tube inside the body or frontal horn of the lateral ventricle. Figure 4 shows the cranial tube with inappropriate location and complications: long crossing tube, short tube, and extradural hematoma.

## Discussion

The placement of a ventriculo-peritoneal shunt is the current standard in the treatment of hydrocephalus in spite of high complication rates [8].

Concerning operative complications during the first postoperative month between ultrasound-guided and conventional groups, our results revealed that there were no significant differences between groups, but proximal obstruction which is the commonest complication occurred only in the conventional group (3 cases 10%).

It is believed that obstruction by the choroid plexus is the most common reason for the failure of the cranial catheter of the ventriculoperitoneal shunt [9]. Also, the short proximal tube may lead to obstruction by debris and brain cells in our opinion.

The results of the present study agree with several studies which concluded that shunt catheter obstructions either proximal or distal obstruction are by far the most common cause of shunt malfunction, yet the factors that contribute to this matter remain elusive. Obstruction can occur in the proximal catheter itself or within the valve or within the distal catheter; however, the commonest site of obstruction in most studies is the proximal tube [10–12]. Proximal obstruction at 3 months was 4.2% by conventional method and 1 patient out of 112 patients who operated under navigation view [10]. Multiple studies have compared shunt failure between patients having different types of shunt valves as programmable and non-programmable valves; however, the majority of studies failed to demonstrate an association [13]. Therefore, utilization of trans-fontanel ultrasound for cranial catheter placement in patients with an open fontanel in need of external ventricular drainage, VP shunt insertion, and revision aids in the reduction of proximal catheter failure [11, 14].

In contrast to our results, Whitehead et al. [15] showed that ultrasound-guided cranial catheter insertion by experienced pediatric neurosurgeons resulted in accuracy in only 59.1% of cases. They referred to two main reasons that the ultrasound technique failed to achieve the desired level of accuracy: the first was targeting the wrong area of the ventricular system resulted in an inaccurate location in 7 (10.4%) of 67 patients, and the second was catheter movement between intraoperative and postoperative imaging.

In our study, the intraoperative ultrasound image of the ventricular catheter was obtained while passing through the body of the lateral ventricle with the stylet in place, and if we decided to continue to the frontal horn, we must identify the choroid plexus which appears hyper-echogenic bilaterally and then by tilting the transducer from the coronal plan, we find the pathway to the frontal horn and then advance the tube to the required location and finally remove the stylet; we also take sagittal ultrasound images for confirmation as shown in image 1. After this maneuver, the catheter was attached to the valve.

The use of ultrasound does not cause prolongation or complication of the procedure as the operation time is not prolonged, no risk factor regarding shunt infection. It is accurate as frameless navigation insertion and due to its precise location, there are lower obstruction rate [16]. These were documented in this study.

Our results showed no significant difference between groups regarding follow-up imagining data within the first postoperative 48 ho and after 3 months from surgery except for ventricular tube location in favor of the ultrasound group (*p* = 0.0005 and 0.021). Inadequate location does not mean malfunctioning at the time but may happen later on. Whitehead et al. [15] stated that shunt survival for the 2 groups was similar in their study when compared to conventional and ultrasound-guided maneuvers.

Our results showed that 3 patients from the conventional group needed revisions at the cranial end. There were 2 cases that needed revisions at the peritoneal end in the ultrasound-guided group and one case in the conventional group.

Stone et al. [17] reported the ventriculoperitoneal revision rates in children as 84.5% of patients and 4.7% of patients may require greater than 10 revisions and around 2.66 revisions per patient.

Ventriculoperitoneal shunts are associated with a high rate of malfunction; one study reported revision rates of 32.5% in adults which was significantly higher (78.2%) in children [18]. Shunt malfunction is considered a common neurosurgical problem in patients with ventriculoperitoneal shunts, often leading to frequent and sometimes lengthy hospital stays and multiple revisions throughout their life. Multiple options for reducing shunt malfunctions, such as antibiotic-impregnated catheters, programmable valves, and navigated insertion [19].

In our study, the complicated cases with shunt obstructions which operated again for shunt revisions had been excluded in the follow-up as considered failure of the primary aim.

In general, children with shunt malfunction may be present with the symptoms of headache, vomiting, and drowsiness, but at these young ages, the symptoms can represent many other illnesses [20]. Moreover, Cohen et al. [21] failed to demonstrate any correlation between varieties of symptoms and radiographic evidence of shunt malfunction. However, they found a positive association between bulging fontanels, behavioral change, and shunt revision, suggesting that clinical manifestations alone may provide a valid decision for shunt revision in children with an open fontanel.

A study showed that 25.3% of all CT scans were performed in children less than 2 years of age, suggesting bad radiation exposure could potentially be reduced by clinical assessment and the use of diagnostic scales [22]. We advise the use of ultrasound in follow-up of those patients to avoid radiation exposure, high cost, and long imaging time of CT and MRI.

Regarding shunt infection in our study, there were no significant differences between the groups with an overall rate of 6.7%, and all were treated by conservative treatment. Bastian et al. [23] reported a 15% infection rate in their study on a pediatric ventriculoperitoneal shunt. Pal et al. [10] reported 3.1% shunt infection in their study on pediatrics and adolescent.

## Conclusion

The placement of ventricular catheters in pediatric patients with a patent anterior fontanel under ultrasound guide view is better than the conventional method, and the novel technique of our study gave the ability to reach the frontal horn of the lateral ventricle under real-time safe maneuver from keen point.

## Data Availability

All data that support the findings of this study are available from the Neurosurgery Department at Zagazig University Hospital. Data are however available from the author when requested with permission.
